# Individual differences in emerging adults’ spatial abilities: What role do affective factors play?

**DOI:** 10.1186/s41235-024-00538-w

**Published:** 2024-03-18

**Authors:** Carlos J. Desme, Anthony S. Dick, Timothy B. Hayes, Shannon M. Pruden

**Affiliations:** Department of Psychology, Florida International University, 11200 SW 8th Street, Miami, FL 33199 USA

**Keywords:** Spatial ability, Mental rotation, Perspective taking, Spatial anxiety, Confidence

## Abstract

Spatial ability is defined as a cognitive or intellectual skill used to represent, transform, generate, and recall information of an object or the environment. Individual differences across spatial tasks have been strongly linked to science, technology, engineering, and mathematics (STEM) interest and success. Several variables have been proposed to explain individual differences in spatial ability, including affective factors such as one’s *confidence* and *anxiety*. However, research is lacking on whether affective variables such as *confidence* and *anxiety* relate to individual differences in both a mental rotation task (*MRT*) and a perspective-taking and spatial orientation task (*PTSOT*). Using a sample of 100 college students completing introductory STEM courses, the present study investigated the effects of self-reported *spatial confidence, spatial anxiety,* and *general anxiety* on *MRT* and *PTSOT*. *Spatial confidence,* after controlling for effects of *general anxiety* and *biological sex,* was significantly related to performance on both the *MRT* and *PTSOT*. *Spatial anxiety*, after controlling for effects of *general anxiety* and *biological sex*, was not related to either *PTSOT* or *MRT* scores. Together these findings suggest some affective factors, but not others, contribute to spatial ability performance to a degree that merits advanced investigation in future studies.

## Significance statement

The current study examined why individuals differ in their spatial ability. We use spatial ability daily to complete tasks including packing a suitcase, finding our car in a parking lot, and navigating city streets using GPS. Spatial ability also predicts entry and success in science, technology, engineering, and mathematics disciplines in college. Given the underrepresentation of women and minorities in these disciplines, it is important to understand why individuals differ in their spatial ability, as this may offer a promising route to improving spatial ability via intervention studies. We explored whether one’s own perceived self-confidence in spatial ability and their degree of anxiety reported when completing spatial tasks was related to their performance on two types of spatial tasks, a mental rotation task and a spatial perspective-taking/spatial-orientation task. To solve the mental rotation task, participants had to imagine what tetris-like images looked like from different rotations. To solve the spatial perspective-taking/spatial-orientation task, participants had to imagine standing at one object while facing another object and then point in the direction of a third object. In a sample of 100 college students completing introductory STEM courses, we found that self-confidence in spatial ability was related to their performance on both spatial tasks, such that those who reported higher confidence in their answers performed better on the tasks. We did not find the same relation between spatial anxiety and spatial task performance. Results can be applied to develop interventions or education programs aimed at increasing spatial confidence, as well as spatial ability.

## Introduction

Spatial ability allows us to represent, transform, generate, and recall spatial information such as size, shape, location, distance, and direction (Linn & Petersen, 1985; Sinton et al., 2013). We use spatial abilities in everyday tasks such as navigating city streets, putting together furniture, and packing a suitcase for an upcoming trip. Spatial ability is often used, alongside quantitative and verbal aptitude tests, to identify gifted individuals and individuals who might excel in science, technology, engineering, and mathematics (STEM) careers (McCabe et al., 2020), as individual differences in spatial ability are linked to success in STEM (e.g., Wai et al., 2009). Further, there is a strong association between the development of spatial skills and an increased performance in mathematics, even when accounting for other variables (Verdine et al., 2014). Theories behind these reported relations posit that spatial representations are an important component to the mathematical thinking process required to solve mathematical problems (Mix & Cheng, 2012). Interventions aimed at improving spatial ability have yielded parallel improvements in academic achievement, such as mathematics (Cheng & Mix, 2014; Lowrie et al., 2017), suggesting a potential role for spatial ability to improve STEM outcomes.

Individual differences in spatial ability are present from a young age (Levine et al., 1999) and are maintained throughout adulthood (Hegarty & Waller, 2005). These findings suggest it is critical to understand how spatial ability develops, particularly what factors explain individual differences in spatial ability, as spatial ability is a predictor of later STEM achievement (Stieff & Uttal, 2015; Wai et al., 2009).

The current study examined whether affective factors, like confidence in one’s own spatial abilities and spatial anxiety explain individual differences in two types of dissociable spatial abilities, mental rotation and perspective-taking/spatial orientation (Jansen, 2009). Recent research has demonstrated a potential role for these two affective factors in explaining individual differences in *mental rotation ability* (e.g., Alvarez-Vargas et al., 2020; Arrighi & Hausmann, 2022; Cooke-Simpson & Voyer, 2007; Estes & Felker, 2012; Lawton, 1994; Lyons et al., 2018). Less is known about potential links in affective factors and *perspective-taking/spatial orientation* (though see Lawton, 1994 and Lyons et al., 2018) and few studies have attempted to combine both affective factors into one study to examine their roles in predicting different types of spatial abilities (but see Arrighi & Hausmann, 2022). Should we find a role for affective factors like confidence and spatial anxiety predict individual differences in two types of spatial abilities, this might offer a new promising route to improving spatial ability in intervention studies.

The literature on the role of affective factors on cognitive test performance, such as IQ, suggests an important role for both anxiety and confidence (e.g.., Eysenck et al., 2007; Stankov & Crawford, 1997). Further, domain-specific research in a related domain to spatial cognition, mathematical cognition, also points to a potential role for affective factors on cognitive performance (Ashcraft & Kirk, 2001; Ma, 1999). Some groups have shown that individual differences in mathematical performance are a function of math anxiety (Ashcraft & Faust, 1994; Ashcraft & Kirk, 2001; Faust et al., 1996). Meta-analyses (Ma, 1999) show that mathematics anxiety is correlated with mathematics achievement, with this relation consistently shown across gender, ethnicity, instruments assessing anxiety and even in studies controlling for general or trait anxiety (Daker et al., 2022; Di Lonardo Burr & LeFevre, 2021). Finally, in cross-domain research, spatial anxiety has been shown to mediate sex differences in math anxiety (Delage et al., 2022) and spatial ability has been shown to mediate sex differences in math anxiety (Delage et al., 2022; Maloney et al., 2012). This research suggests both domain-general affect-cognition links and domain-specific affect-cognition links.

### Affective factors explain individual differences in mental rotation

Small-scale spatial ability has been defined as the ability to mentally transform representations of small shapes or parts of shapes (Hegarty et al., 2006). Sometimes referred to as allocentric spatial transformations (Wang et al., 2014) or object-based transformations (Zacks et al., 2000), these transformations can be accomplished by coding an object’s location relative to other objects, regardless of the viewer’s position. Mental rotation, one example of a small-scale spatial ability, is defined as the ability to rotate 2-dimensional and 3-dimensional objects in space using one’s mind (Voyer et al., 1995). Individual differences in mental rotation ability have been linked to not only STEM entry, but also attaining success within such domains (Wai et al., 2009). It is also established that males consistently outperform females on mental rotation tasks (Voyer et al., 1995), which may be one explanation for the underrepresentation of females in STEM fields.

Two affective factors appear to explain observable individual differences in mental rotation including spatial confidence (Arrighi & Hausmann, 2022; Cooke-Simpson & Voyer, 2007; Desme et al., 2024; Estes & Felker, 2012) and spatial anxiety (Alvarez-Vargas et al., 2020; Arrighi & Hausmann, 2022; Lawton, 1994; Lyons et al., 2018; Ramirez et al., 2013). Below, we review the existing evidence that both confidence and spatial anxiety explain individual differences in mental rotation in emerging adulthood.

Confidence in one’s own ability has been linked to other cognitive abilities including mathematics ability (e.g., Casey et al., 1997), a domain tightly linked to spatial ability (Lubinski & Benbow, 1992; Mix & Cheng, 2012). Within the study of spatial ability, confidence has also been associated with mental rotation performance (Cooke-Simpson & Voyer, 2007; Estes & Felker, 2012). Cooke-Simpson and Voyer (2007) were among the first to examine the role of confidence in mental rotation performance. They surmised that participants who were guessing were more likely to be less confident in their answers, so they examined the role of confidence as an explanation for overall performance. Using a sample of undergraduate students, they found that a large correlation (*r* = 0.685) between MRT and average confidence rating. Similarly, in a series of 4 experiments Estes and Felker (2012) explored whether confidence was related to individual and sex differences in mental rotation performance. They found that males were not only more confident than females in their own mental rotation abilities, but that individuals who rated themselves as more confident in the accuracy of their responses were in fact more accurate on a classic mental rotation task. These two foundational studies established there may be an important role for confidence in explaining individual differences in mental rotation.

Spatial anxiety, a domain-specific anxiety, has been linked to both individual and sex differences in mental rotation across numerous studies (e.g., Alvarez-Vargas et al., 2020; Lawton, 1994; Lyons et al., 2018; Ramirez et al., 2013). Lawton (1994) developed a measure of spatial anxiety that asked participants to indicate their level of anxiety on a 5-point Likert scale to situations that required navigational or wayfinding skill. Women were more likely to report higher levels of spatial anxiety, which also in turn was related to their preferred strategy use for navigating or wayfinding. This groundbreaking study was limited in that it did not seek to examine directly whether individual or sex differences in mental rotation were directly related to spatial anxiety.

More recently, Alvarez-Vargas and colleagues (2020) explored whether reported sex differences in mental rotation could be explained by different types of spatial anxiety including navigation and mental rotation anxiety. They modified Lawton’s (1994) spatial anxiety questionnaire to include not only questions related to navigation and wayfinding anxiety but also questions related to mental rotation anxiety like solving puzzles, playing Tetris, and packing a suitcase. In a large sample of over 500 emerging adults, they found that mental rotation anxiety was the only significant mediator of sex differences in mental rotation performance, suggesting a unique role for not only navigation anxiety but mental rotation anxiety in explaining individual differences in mental rotation. Finally, Lyons et al. (2018) created their own new spatial anxiety scale to assess whether different kinds of spatial anxiety, including navigation and mental-manipulation anxiety, are related to spatial ability. Not only did they find evidence for three different kinds of spatial anxieties (i.e., imagery, navigation, and mental-manipulation) using a data-driven exploratory factor analysis, but critically they found support for specific relations between mental-manipulation anxiety and mental rotation performance, as well as self-reported mental rotation ability ratings. These findings highlight again the potential role that not only spatial anxiety, broadly defined plays in mental rotation performance but also points to a unique role that mental rotation anxiety, a specific sub-type of spatial anxiety might play in mental rotation ability.

In recent years there have been attempts to combine multiple affective factors into one study to examine their role in mental rotation (Arrighi & Hausmann, 2022; Desme et al., 2024). Desme and colleagues sought to examine the direct and indirect roles of spatial confidence, spatial anxiety, and prior engagement in spatial toys and activities in childhood as mediators of sex differences in mental rotation (Desme et al., 2024). A battery of measures was administered to 464 college students taking psychology courses or majoring in psychology. Students were assessed on their mental rotation performance, their confidence in their mental rotation performance, their level of spatial anxiety, and their engagement in spatial activities as youth. Males reported less spatial anxiety (and general anxiety), engaged with more spatial activities, and were more confident in their mental rotation performance. A path analysis demonstrated participant biological sex predicted engagement in some sex-typed spatial activities, which in turn predicted level of spatial anxiety and amount of spatial confidence, and which finally predicted individual differences in MRT scores. Based on these results, it was inferred that males engaged more frequently with spatial activities, leading to decreased spatial anxiety, increased confidence on MRT and ultimately better MRT outcomes than females. Together, these findings suggest an important role for both early experiences as well as affective factors, confidence, and spatial anxiety in explaining reported sex differences in mental rotation ability.

Finally, Arrighi and Hausmann (2022) explored whether confidence and spatial anxiety mediate sex differences in mental rotation using two different types of mental rotation tasks, the revised Vandenburg and Kuse MRT (1978) involving detailed 2D drawings of 3D cube figures and a 2D mirror pictures task. Double-mediation models revealed that both confidence and spatial anxiety partially mediated the reported sex differences in both mental rotation tasks. Like Desme et al. (2024), these results point to the important role that both confidence and spatial anxiety may play in explaining the variability in skill seen in mental rotation tasks. Both Desme et al. (2024) and Arrighi and Hausmann (2022) focused explicitly on explaining reported sex differences in the literature. We aim to identify how these affective factors relate to individual differences in mental rotation ability, controlling for participant biological sex, to identify other potential participant characteristics that explain variability performance.

Given the prior literature on both spatial confidence and spatial anxiety and relations to individual differences in mental rotation, we aim to replicate prior work in the present study and show that confidence and spatial anxiety broadly defined, as well as the more narrowly defined small-scale (or mental rotation) spatial anxiety, are related to individual differences in mental rotation performance. In addition, we extend this line of research by examining the relative contributions of *both* affective factors in one model in explaining individual differences in mental rotation.

### Affective factors explain individual differences in perspective-taking/spatial orientation

Large-scale spatial ability has been defined as the ability to engage in egocentric spatial transformations (e.g., mental rotation along body axis; Hegarty & Waller, 2004) and require that participants view the larger environment from different perspectives even when the relations between objects remain the same. This ability is dissociable from small-scale spatial ability (Hegarty et al., 2006) and has been referred to by a number of different labels including perspective-taking (Hegarty & Waller, 2004) and spatial orientation (Kozhevnikov & Hegarty, 2001). Perspective-taking, a type of large-scale spatial ability, is the ability to imagine objects in an environment from an alternative or different point of view (Hegarty & Waller, 2004; Jansen, 2009). In one of the most widely used perspective-taking tasks, known as the perspective-taking/spatial orientation tasks (PTSOT; Hegarty & Waller, 2004), participants are asked to imagine themselves in a different orientation. This task requires that participants imagine being located at a particular object among an array of objects, and while facing a second object in the array indicate the direction of a third object. Moderate to large individual differences in this ability have been documented (Tarampi et al., 2016). Little is known about the affective factors, like confidence and spatial anxiety, that explain individual differences in this spatial ability. Thus, the second aim of the current study is to examine whether the affective factors that predict individual differences in mental rotation also explain individual differences in perspective-taking/spatial reorientation, a dissociable spatial ability (Hegarty & Waller, 2004). There is some research linking navigation ability to these affective factors. We review this literature here with the aim of showing that there is reason to examine how affective factors are related to other types of spatial abilities beyond mental rotation.

There are mixed findings with respect to the relation between confidence and spatial abilities. For example, Picucci et al. (2011) found no correlation between accuracy on a computer-adapted spatial reorientation task and participant confidence. Participants were asked to remember where an object was hidden in a rectangular or square room that contained either or both geometric cues (e.g., length of walls) and landmark cues (e.g., color of a wall). Participants were also asked to rate how confident they were in their spatial reorientation ability. While no relation was found between spatial reorientation accuracy and confidence in this study, others have hypothesized that links should be found between confidence and performance in spatial reorientation (e.g., Nardi et al., 2013).

Unlike research on confidence, research does suggest a link between spatial anxiety and a variety of spatial abilities beyond mental rotation (He & Hegarty, 2020; Hund & Minarik, 2006; Lyons et al., 2018). There are findings that adults who are more spatially anxious make more navigation errors when following route directions using a scale model of a town (Hund & Minarik, 2006). Further, as mentioned previously when reviewing the mental rotation literature, Lyons and colleagues (2018) had developed a spatial anxiety measure that included not only items asking about mental manipulation anxiety, but also about navigation anxiety. Using this measure, they found that adults with higher levels of navigation anxiety show a decrease in accuracy on a map navigation task where they had to imagine walking along a dotted path while making judgements about whether turns on that path were labeled correctly or incorrectly (e.g., right/left). More recently, He and Hegarty (2020) examined explanations for individual differences in navigation ability, with a focus on how motivation and emotional disposition relates to navigation and GPS dependency. Using Lawton’s (1994) 8-item spatial anxiety measure, they showed that individuals with lower levels of navigation and wayfinding anxiety had higher scores on a self-report sense of direction measure and scored higher on a perspective task like the one we use in the current study.

Together, prior literature does point to a *potential* link between affective factors and individual differences in perspective-taking. Yet, more research is needed to explore potential relations between different types of spatial abilities and links to both confidence and spatial anxiety within a single study.

## The present study

The current study seeks to understand how two affective factors, spatial confidence and spatial anxiety, relate to individual differences in two dissociably different spatial abilities, mental rotation and perspective-taking/spatial orientation. We aimed to both replicate and expand upon previous research (Alvarez-Vargas et al., 2020; Arrighi & Hausmann, 2022; Cooke-Simpson & Voyer, 2007; Desme et al., 2024; Estes & Felker, 2012; He & Hegarty, 2020; Lawton, 1994; Lyons et al., 2018; Munion et al., 2019; Picucci et al., 2011; Ramirez et al., 2013) by examining individual differences across two different types of spatial abilities, mental rotation, and perspective-taking/spatial orientation. There is little research about whether affective factors such as spatial confidence and spatial anxiety relate to other types of spatial ability, beyond that of mental rotation, to abilities like perspective-taking/spatial orientation (Linn & Petersen, 1985). Specifically, in the present study, we examined the relations between *two* types of affective factors, spatial anxiety and confidence, and *two* types of spatial ability, mental rotation and perspective-taking/spatial orientation, in a sample of undergraduate students completing introductory STEM courses.

We have two main aims in the current study: (1) Do the affective factors, spatial confidence and spatial anxiety, relate to individual differences in mental rotation? (2) Do the affective factors, spatial confidence and spatial anxiety, relate to individual differences in perspective-taking/spatial orientation? We also have more exploratory aims where we look at whether specific types of anxiety (e.g., mental rotation as small-scale anxiety and navigation anxiety as large-scale anxiety) relate specifically to mental rotation and perspective-taking/spatial orientation, respectively. We expected to replicate prior literature showing that both confidence and spatial anxiety significantly relate to mental rotation performance. We also predicted the same for perspective-taking/spatial orientation despite mixed reports and mere speculations in the literature. Finally, we predicted that small-scale anxiety would be related to mental rotation ability, but not perspective-taking/spatial orientation ability, but that large-scale anxiety will be related to perspective-taking/spatial orientation ability and not mental rotation ability.

## Methods

### Participants

Data were gathered on 100 undergraduate students attending a public 4-year university in the Southeastern USA. Participants were asked to self-report their biological sex at birth. Biological sex was treated as a covariate in all analyses. A total of 34 males and 66 females completed the survey. Participants’ average age was 24 years (*Range* = 22 to 28 years). The ratio of males to females in our sample roughly aligned with the number of undergraduate males (~ 26.7%) and females (~ 73.3%) newly enrolled in the college from which we recruited. Participants varied in their racial identity with 72% identifying as White/Caucasian, 9% identifying as black/African American, 9% identifying as Asian, and 5% identifying as two or more races or some other race.

To be eligible to participate, participants must have completed one of the following introductory STEM courses: biology 1, calculus 1, chemistry 1, and/or physics 1. The pool of participants represented 14 different undergraduate majors with 35% majoring in psychology, 25% majoring in engineering, 18% majoring in biology, 7% majoring in chemistry, 4% majoring in computer science, 4% majoring in nursing, 3% majoring in physics, 2% majoring in geology, and 1% majoring in anthropology, education, economics, information technology, mathematics and mass communications. Participants were recruited by contacting instructors teaching each of the listed courses above and by contacting department advisors in biology, mathematics, chemistry, and physics via e-mail. Instructors and advisors were provided with a flyer of the online study to distribute to students in their courses or that they were advising. Participants received a $10 gift card after completion of the survey.

### Measures and procedure

Participants completed the following measures in a fixed order in a fully online survey: (1) a consent form; (2) a 24-item *Mental Rotation Test (MRT*); (3) a 12-item *Perspective Taking/Spatial Orientation Task (PTSOT)*; (4) a total of 60 spatial confidence questions, of which 48 questions were about confidence in answers on the *MRT* and 12 questions were about confidence in answers on the *PTSOT*; (5) an 21-item *Modified Spatial Anxiety Scale* (Alvarez et al., 2020), with 8 items asking about large-scale spatial anxiety and 13 items asking about small-scale spatial anxiety; and (6) a 20-item *State-Trait-Anxiety Inventory—Trait Subscale (STAI-T)* which served as a measure of general anxiety. Self-reported confidence and anxiety were the last questions to be asked as we did not want to inadvertently induce or manipulate levels of confidence or anxiety prior to completing the outcomes of interest, the *MRT* and *PTSOT*. A fixed order of measures was adopted as we wanted to: (1) ensure participants could judge their confidence after each item instead at the end of the spatial task to reduce potential memory load; (2) reduce the risk of stereotype threat by administering the spatial anxiety questionnaire only after the completion of the spatial tasks; and (3) minimize potential data loss of our main measures of interest by giving all other measures such as general anxiety and demographics questions at the end of the study.

#### Mental rotation test (MRT)

**Fig. 1:**
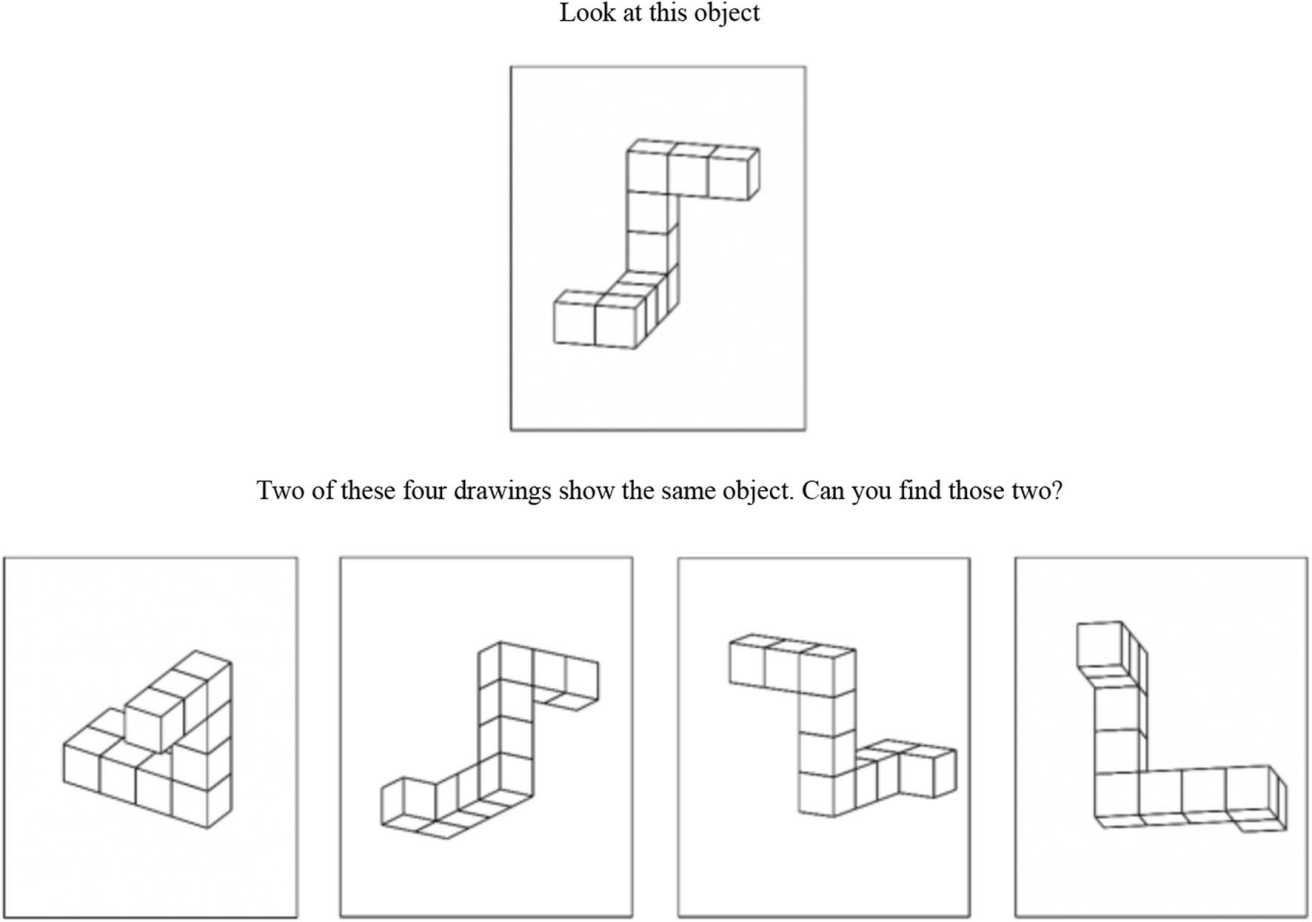
Sample item from *MRT*. Participants were instructed to select two of the four options they believed were identical to the target figure

The revised Vandenburg and Kuse *Mental Rotation Test* (*MRT*; Peters et al., 1995; Vandenburg & Kuse, 1978) was administered to participants. This task consisted of 4 training trials followed by 24 test trials. The task was untimed as prior literature suggests that timed measures can potentially increase anxiety and induce stereotype threat (Schmader & Johns, 2003). Feedback on whether the participant was correct was only provided during the four training trials to make sure participants understood the task instructions. No feedback was provided to the participant during the test trials. During each trial, participants were shown a 3D target figure and four rotated test figures (Fig. 1). Two test figures were rotated along the x- and y-axis, and if rotated could be matched to the target figure. The other two test figures were rotated *mirror images* of the target figure and could never be rotated to match the target figure. The participant was instructed to select two of the four options they believed were identical to the target figure using the following language: “Two of the 4 drawings show the same object. Can you find those two?”. Participants were required to select both correct answers on a test trial to receive a point for that trial. This yielded a total raw score of up to 24 points for the variable, *MRT*. The *MRT* had a high degree of reliability, and was deemed acceptable (Taber, K.S., 2018*),* with a reported Cronbach’s alpha of α = 0.80.

#### Perspective-taking spatial orientation task (PTSOT)

An adapted version of the *Perspective-Taking Spatial Orientation Task* (*PTSOT*; Hegarty & Waller, 2004; Kozhevnikov & Hegarty, 2001) was administered next. This task has been shown to have a high degree of internal reliability (0.83) and validity as a spatial orientation task according to Kozhevnikov and Hegarty (2001). It also been shown to separable from spatial visualization, spatial relation and mental rotation tasks (Hegarty & Waller, 2004). The *PTSOT* requires individuals to imagine themselves at a particular place and orientation in an environment while determining the direction of travel to another place within that very same environment. Lastly, this task has been shown to predict performance in a wide variety of spatial cognition tasks including sense of direction and route planning (e.g., Hegarty & Waller, 2004).

**Fig. 2:**
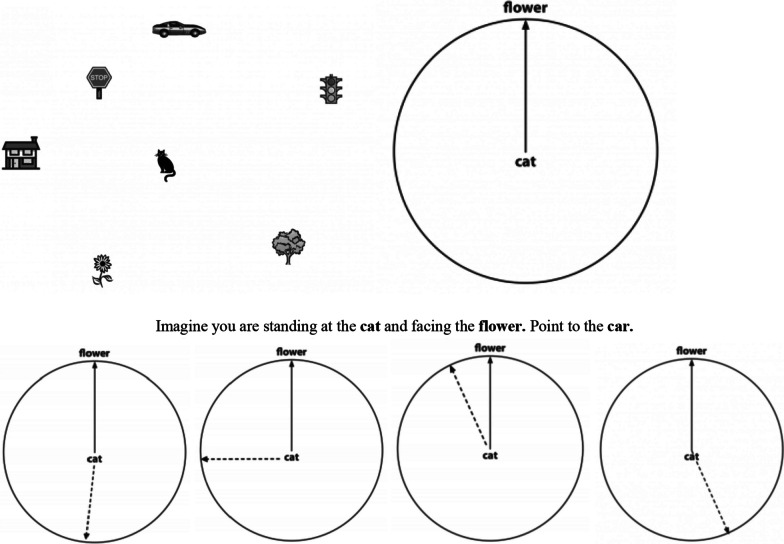
Sample item from *PTSOT.* Participants were shown an array of 7 objects and were asked to imagine standing at one object while facing another object and then point in the direction of a third object

We adapted this task to be given on a computer and with four multiple-choice test items rather than asking participants to draw a line as they were instructed to do in the original *PTSOT* task. Like the *MRT*, this task was untimed so as to reduce the likelihood of increased anxiety or stereotype threat. Feedback was provided for only the training trials and not the test trials. Two training trials and 12 test trials were administered. Participants were shown an array of 7 objects and were asked to imagine standing at one object while facing another object and then point in the direction of a third object (e.g., “Imagine you are standing at the cat and facing the flower. Point to the car; Fig. 2). They were also shown a main target circle that had an upright line drawn between the first (e.g., cat) and second object (e.g., flower). Participants were then asked to indicate which of the four multiple-choice items depicted where they would be pointing when pointing to the third object (e.g., car). Only one answer was possible for each test trial, and thus, the total maximum score on the PTSOT was 12. The *PTSOT* had an acceptable degree of reliability with a reported Cronbach’s alpha of α = 0.79.

#### Spatial confidence

Spatial confidence was measured via self-report of confidence after each item for both the *MRT* and *PTSOT*. To assess confidence, we chose to gather item-level confidence data (e.g., Jonsonn & Allwood, 2003; Lundeberg et al., 1994) rather than confidence across a whole measure. Assessing confidence at the item-level meant the participant had time to reflect on each item instead of remembering at the end of the spatial task how they generally felt about their performance. This methodological choice to test item-level confidence reduced potential memory interference such that participants were not asked to recall how they felt across the whole task nor were they likely to show recency effects with the last few items being those that they remembered best.

A total of 60 spatial confidence questions were administered, of which 48 questions were about confidence in answers on the *MRT* and 12 questions were about confidence in answers on the *PTSOT.* Each test trial on the *MRT* had two spatial confidence questions, each corresponding to their first and second selections. The language used to evaluate confidence in *MRT* choices was as follows, “How confident are you in your [1st, 2nd] choice being the correct one?” For *PTSOT*, only a single spatial confidence question was asked (“How confident are you in your choice being the correct one?”), as there was only one correct answer for each item. A 7-point Likert scale was used with the following labels, “Not at All Confident” [0], “Not Very Confident” [1], “Somewhat Unconfident” [2], “Neither Confident nor Unconfident” [3], “Somewhat Confident” [4], “Very Confident” [5], and “Extremely Confident” [6]. An average spatial confidence score collapsed across both tasks was calculated for each participant. Our item-level spatial confidence measure had an acceptable degree of reliability with a reported Cronbach’s alpha of α = 0.63.

#### Spatial anxiety scale (M-SAS)

The modified *Spatial Anxiety Scale* (*M-SAS*; Alvarez et al., 2020; Lawton, 1994) was administered to participants. There was a total of 21 items, with 8 items asking about *large-scale spatial anxiety* and 13 items asking about *small-scale spatial anxiety* (see Table 1 for items). Items about large-scale spatial anxiety were asked before items about small-scale spatial anxiety. For each item, participants were asked to report the degree of anxiety they felt during certain scenarios. A 4-point Likert scale was used with the following labels, “Not at All” [0], “Mildly (It would not bother me much)” [1], “Moderately (It would be very unpleasant, but I could tolerate it)” [2], “Severely (I could barely tolerate it)” [3]. Three variables were calculated: (1) *Spatial Anxiety* which reflected average anxiety scores across all 21 items; (2) *large-scale anxiety* which reflected average anxiety scores across 8 large-scale items; and (3) *small-scale anxiety* which reflected average anxiety scores across 13 small-scale items. The *M-SAS* had a high degree of reliability with a reported Cronbach’s alpha of α = 0.87.

**Table 1:** Modified Spatial Anxiety Scale (M-SAS; Alvarez et al., 2020)

1. Leaving a store that you have been to for the first time and deciding which way to turn to get to a destination. *
2. Finding your way out of a complex arrangement of offices that you have visited for the first time. *
3. Pointing in the direction of a place outside that someone wants to get to and has asked you for directions, when you are in a windowless room. *
4. Locating your car in a very large parking lot or parking garage. *
5. Trying a new route that you think will be a shortcut, without the benefit of the map. *
6. Finding your way back to a familiar area after realizing you have made a wrong turn and become lost while driving. *
7. Finding your way around in an unfamiliar mall. *
8. Finding your way to an appointment in an unfamiliar city or town. *
9. Constructing a tent at the beach
10. Following origami paper folding instructions
11. Building a Lego Architecture® Empire State building using the instructions
12. Playing Tetris®
13. Folding flattened cardboard into a gift box by following the folds/creases
14. Untangling severely tangled headphone cords
15. Building a 6-drawer dresser from IKEA by following the diagram
16. Solving a 1000-piece puzzle
17. Constructing a model house using Legos using only an image of the end product
18. Packing a trunk with limited space and a lot of objects
19. Packing a carry-on suitcase with many belongings
20. Moving all of your furniture from a larger space into a smaller space
21. Hanging up several pictures, frames, or decals on a wall

*Indicates items asking about large-scale anxiety, all other items asked about small-scale anxiety)

#### General anxiety scale

The last measure given was the 20-item *State-Trait Anxiety Inventory—Trait Subscale* (*STAI-T* Spielberger et al., 1970*;*), a measure of general anxiety (Table 2). This was as a control for general anxiety to ensure our results were unique to spatial anxiety rather than general anxiety. Participants were asked to report their agreement with the statement using a 4-point Likert scale with following labels, “Not at All” [0], “Somewhat” [1], “Moderately So” [2], “Very Much” [3]. Items that were positive statements (e.g., “I feel pleasant”) were reverse coded to align with items that were negative statements (e.g., “I feel like a failure”). A higher score across all items indicated an individual with greater general anxiety. An average score for *general anxiety* was computed. A reported Cronbach’s alpha of α = 0.86 indicates high reliability.

**Table 2:** State-trait anxiety inventory–trait subscale (STAI-T)

1. I feel pleasant
2. I feel nervous and restless
3. I feel satisfied with myself
4. I wish I could be as happy as others seem to be
5. I feel like a failure
6. I feel rested
7. I am “calm, cool, and collected”
8. I feel that difficulties are piling up so that I cannot overcome them
9. I worry too much over something that really doesn’t matter
10. I am happy
11. I have disturbing thoughts
12. I lack self-confidence
13. I feel secure
14. I make decisions easily
15. I feel inadequate
16. I am content
17. Some unimportant thought runs through my mind and bothers me
18. I take disappointments so keenly that I can’t put them out of my mind
19. I am a steady person
20. I get in a state of tension or turmoil as I think over my recent concerns and interests

## Results

R software (R Core Team, 2021) was used to analyze all data gathered. To mitigate the effect of extreme outliers and retain power by not eliminating outliers, we opted to Winsorize our main variables of interest. This technique down weights the influence of outliers but allows us to retain the full power of our sample size. Psychometric properties of the variables and descriptive statistics were derived and are recorded below. To examine associations between *spatial confidence, spatial anxiety, general anxiety* and our dependent measures, *MRT* and *PTSOT*, we entered these variables into structural equation models. All models were fully saturated, as noted below with each analysis (i.e., the predicted covariance matrix perfectly reproduces the observed covariance matrix).

### Descriptive statistics and correlations

Descriptive statistics, including means, standard deviations, and ranges, were computed for all variables with the goal of identifying any issues with ceiling or floor effects. A comprehensive summary displaying descriptive statistics information of the variables is depicted in the table below (Table 3). All variables showed sufficient variability and no ceiling or floor effects were evident. Pearson correlations were performed to estimate the relation and direction, either positive or negative, between all variables (Table 4). No correlation values exceeded 0.80, the threshold that might indicate multicollinearity between measures.

**Table 3:** Descriptive statistics

Variable	*M*	*SD*	Min	Max
*1. MRT*	15.83	7.15	1.00	24.00
*2. PTSOT*	8.02	3.53	0.00	12.00
*3. Spatial confidence*	4.53	1.16	1.44	6.00
*4. Spatial anxiety*	1.00	0.50	0.00	2.45
*5. Large-scale anxiety*	1.20	0.67	0.00	2.88
*6. Small-scale anxiety*	0.80	0.55	0.00	2.54
*7. General anxiety*	1.39	0.24	0.90	1.91

Sex was dummy coded as males = 2 and females = 1

**Table 4:** Means, standard deviations, and correlations with confidence intervals

Variable	*M*	*SD*	1	2	3	4	5	6	7
1. *Sex*	1.34	0.48							
2. *MRT*	15.83	7.15	.27**						
			[.08, .44]						
3. *PTSOT*	8.02	3.53	.28**	.65**					
			[.09, .45]	[.51, .75]					
4. *Spatial Confidence*	4.53	1.16	.40**	.60**	.60**				
			[.23, .56]	[.46, .71]	[.46, .72]				
5. *Spatial Anxiety*	1.00	0.50	− .29**	− .09	− .25*	− .31**			
			[− .46, − .10]	[− .29, .10]	[− .42, − .05]	[− .47, − .12]			
6. *Large-Scale Anxiety*	1.20	0.67	.03	.01	.15	.07	− .03		
			[− .16, .23]	[− .18, .21]	[− .05, .34]	[− .13, .26]	[− .22, .17]		
7. *Small-Scale Anxiety*	0.80	0.55	.07	.05	.03	.04	.03	.55**	
			[− .13, .26]	[− .15, .24]	[− .16, .23]	[− .16, .23]	[− .17, .23]	[.40, .67]	
8. *General Anxiety*	1.39	0.24	− .03	.11	.02	.00	.30**	.13	.09
			[− .23, .17]	[− .09, .30]	[− .18, .21]	[− .20, .19]	[.10, .46]	[− .07, .31]	[− .11, .28]

Values in square brackets indicate the 95% confidence interval for each correlation. Confidence intervals represent the possible range of population correlations that may have caused the correlation (Cumming, 2014). *p < .05. **p < .01

**Fig. 3:**
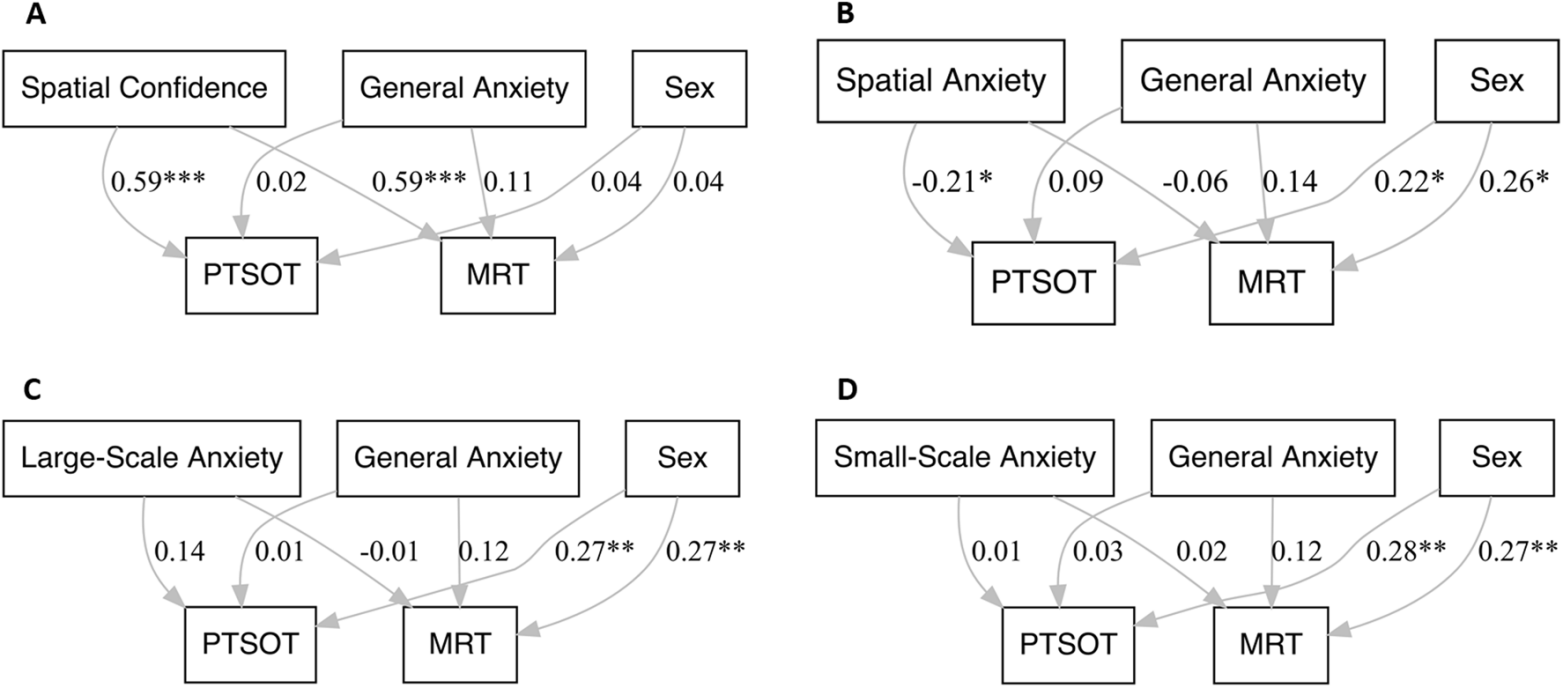
Models testing relations between spatial confidence (**A**), spatial anxiety (**B**), large-scale anxiety (**C**), small-scale anxiety (**D**) and MRT and PTSOT as outcome variables, with covariates. Path coefficients indicate the standardized slope estimate. Statistically significant associations are marked next to the path coefficient with *. Confidence intervals for significant estimates are reported in the text. *** *p* < .001; ** *p* < .01; * *p* < .05

**Fig. 4:**
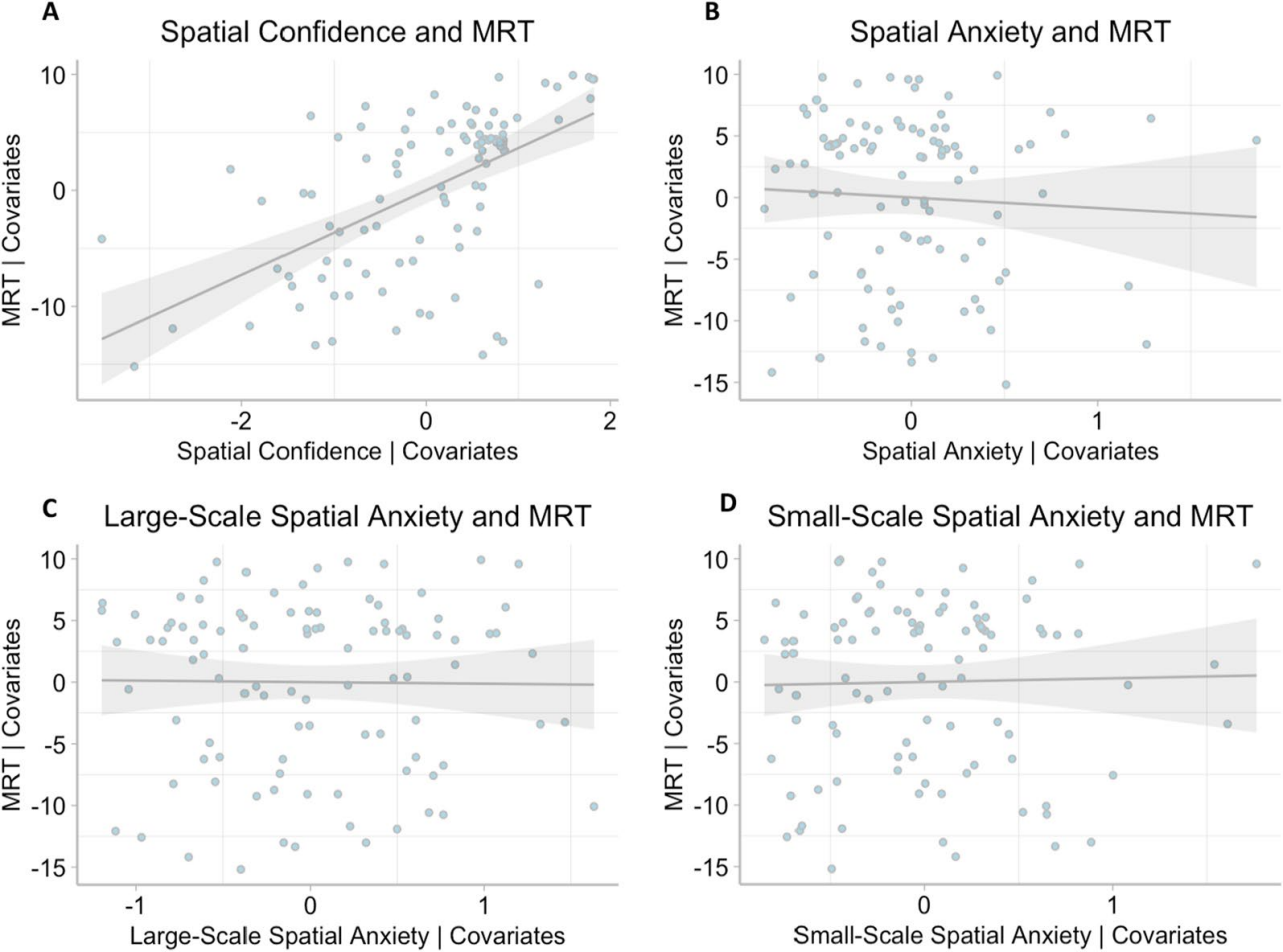
Scatterplots between spatial confidence (**A**)***, spatial anxiety (**B**), large-scale anxiety (**C**), small-scale anxiety (**D**) and MRT, with covariates. *** *p* < .001

**Fig. 5:**
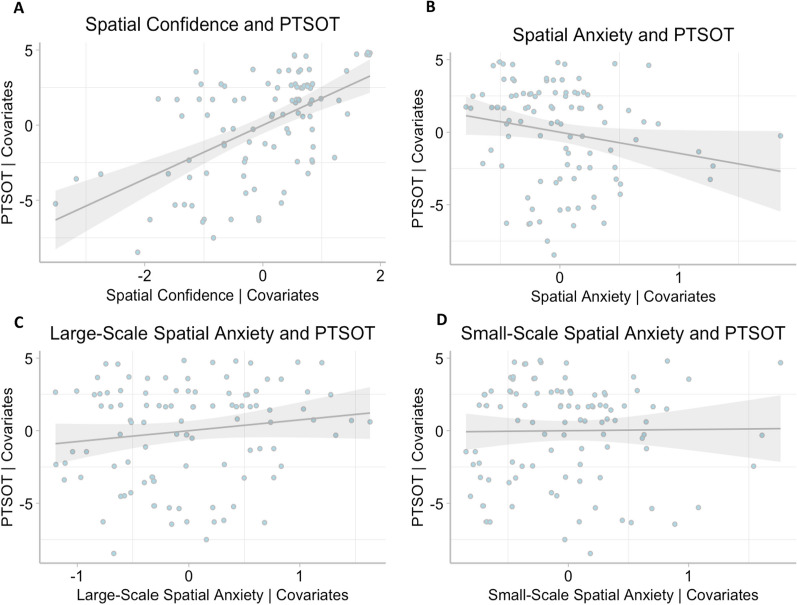
Scatterplots between spatial confidence (**A**)**, spatial anxiety (**B**)**, large-scale anxiety (**C**), small-scale anxiety (**D**) and PTSOT, with covariates. ** *p* < .01

Four SEM models were conducted to examine our aims. Model 1 specified *MRT* and *PTSOT* as outcomes in the same model. *Spatial confidence*, *participant biological sex*, and *general anxiety* were entered as predictors. We first examined fit of the model to the data. As expected, the perfectly saturated model displayed an excellent fit to the observed data, with a chi-square (χ^2^) statistic of 0 (*df* = 0). Other fit indices, including RMSEA, CFI, TLI, and SRMR, also indicated a perfect fit, with values of 0 for RMSEA and SRMR, and 1 for CFI and TLI. These results are expected for a perfectly saturated model and allow us to examine the path coefficients. The results of the analysis of the path coefficients showed that *MRT* was significantly predicted by *spatial confidence*, *β* = 0.59, *z* = 6.82, *p* < 0.001, 95% CI [0.42, 0.76] and *PTSOT* was significantly predicted by *spatial confidence*, *β* = 0.59, *z* = 6.77, *p* < 0.001, 95% CI [0.42, 0.76] (see Fig. 3A), after controlling for *general anxiety* and *participant biological sex*. The significant relation between *spatial confidence* and *MRT,* controlling for our covariates, can be visualized in Fig. 4A, while the significant relation between spatial confidence and PTSPOT controlling for the covariates can be seen in Fig. 5A.

In Model 2, *MRT* and *PTSOT* were again outcomes, and *spatial anxiety*, *participant biological sex*, and *general anxiety* were entered as predictors. As expected, the perfectly saturated model displayed an excellent fit to the observed data, with a chi-square (χ^2^) statistic of 0 (*df* = 0). Other fit indices, including RMSEA, CFI, TLI, and SRMR, also indicated a perfect fit, with values of 0 for RMSEA and SRMR, and 1 for CFI and TLI. After controlling for *general anxiety and participant biological sex*, *spatial anxiety* did not predict *MRT*, *β* =  − 0.06, *z* =  − 0.58, *p* = 0.57, 95% CI [-0.27, 0.15], but it did predict *PTSOT*, *β* =  − 0.21, *z* =  − 2.01, *p* = 0.045, 95% CI [− 0.41, − 0.005] (see Fig. 3B). Figure 4B shows the lack of relation seen between *spatial anxiety* and *MRT*, while controlling for the covariates. We can see the significant relation between *spatial anxiety* and *PTSOT*, after controlling for the covariates, in Fig. 5B.

In Model 3, *MRT* and *PTSOT* were again outcomes*, and large-scale anxiety* was entered as a predictor, with *general anxiety* and *participant biological sex* entered as covariates. This model displayed an excellent fit to the observed data, with a chi-square (χ^2^) statistic of 0 (*df* = 0). Other fit indices, including RMSEA, CFI, TLI, and SRMR, also indicated a perfect fit, with values of 0 for RMSEA and SRMR, and 1 for CFI and TLI. *large-scale anxiety* did not relate to *MRT, β* =  − 0.01, *z* =  − 0.12, *p* = 0.90, 95% CI [-0.20, 0.18]. *large-scale anxiety* also did not relate to *PTSOT* (*β* = 0.14, *z* = 1.47, *p* = 0.14, 95% CI [-0.05, 0.33] (see Fig. 3C). Figure 4C shows the lack of relation between *large-scale anxiety* and *MRT,* and Fig. 5C also shows the null finding between *large-scale anxiety* and *PTSOT.* Both scatterplots depict these relations while controlling for covariates.

In our final model, Model 4, *MRT* and *PTSOT* were our outcome variables*, and small-scale anxiety* (Model 4) was entered as a predictor, with *general anxiety* and *participant biological sex* entered as covariates. Model 4 showed an excellent fit to observed data, with a chi-square (χ^2^) statistic of 0 (*df* = 0). Our other fit indices, RMSEA, CFI, TLI, and SRMR, also showed a perfect fit, with values of 0 for RMSEA and SRMR, and 1 for CFI and TLI. *small-scale anxiety* did not relate to either *MRT, β* = 0.02, *z* = 0.24, *p* = 0.81, 95% CI [-0.17, 0.21], nor *PTSOT*, *β* = 0.01, *z* = 0.13, *p* = 0.90, 95% CI [-0.18, 0.20 (see Fig. 3D). The lack of relation between *small-scale anxiety* and *MRT,* controlling for our covariates, can be seen in Fig. 4D, while the null funding between *small-scale anxiety* and *PTSOT*, controlling for the covariates can be seen in Fig. 5D.

## Discussion

The aims of the current study were to address the following questions: (1) Do the affective factors, spatial confidence and spatial anxiety, relate to individual differences in mental rotation? (2) Do the affective factors, spatial confidence and spatial anxiety, relate to individual differences in perspective-taking/spatial orientation? In addition, we examined in a more exploratory fashion whether large-scale and small-scale spatial anxiety relate more specifically to *PTSOT* and *MRT* spatial ability, respectively.

We expected to replicate previous findings showing links between individual differences in affective factors like spatial confidence and spatial anxiety and mental rotation performance (Alvarez-Vargas et al., 2020; Arrighi & Hausmann, 2022; Cooke-Simpson & Voyer, 2007; Desme et al., 2024; Estes & Felker, 2012; Lawton, 1994; Lyons et al., 2018; Ramirez et al., 2013). We replicated findings that spatial confidence is related to individual difference in mental rotation, showing that participants who are more confident do better on mental rotation. We also predicted that we would replicate results showing that spatial anxiety relates to mental rotation scores, while expanding this research to look specifically at relations between different types of spatial anxiety, small- and large-scale anxiety and mental rotation performance. Surprisingly, we were not able to replicate reported links between spatial anxiety and mental rotation scores. While there were correlations between spatial anxiety and mental rotation, when we controlled for effects of general anxiety and biological sex in our regression models, we found neither spatial anxiety, nor small- or large-scale anxiety predicted individual differences in mental rotation scores.

With respect to perspective-taking/spatial orientation, we aimed to extend prior research findings in some new ways. First, findings on relations between spatial confidence and perspective-taking or spatial orientation are mixed with some finding no relation (Picucci et al., 2011), but others positing there should be one (Nardi et al, 2013). We sought to find evidence that there is a relation between spatial confidence and perspective-taking ability. We found that spatial confidence was related to individual differences in perspective-taking/spatial orientation performance such that those who were higher in confidence in their responses were also more accurate in their perspective-taking/spatial orientation performance. Second, we aimed to build on prior findings showing that spatial anxiety is related to other types of spatial ability beyond mental rotation like navigation and wayfinding ability (He & Hegarty, 2020; Hund & Minarik, 2006; Lyons et al., 2018). We predicted that spatial anxiety would explain variability in perspective-taking/spatial orientation ability given the high degree of overlap between navigation ability and perspective-taking. In addition, we explored whether it is large-scale spatial anxiety only or if small-scale spatial anxiety similarly relates to perspective-taking ability. Contrary to what we predicted, we did not find evidence for a relation between spatial anxiety and perspective-taking/spatial orientation after we had controlled for general anxiety and biological sex of the participant. We similarly did not find any evidence for either large- or small-scale spatial anxiety relating to perspective-taking scores in more exploratory analyses.

We found that our adapted computer-based, multiple-choice PTSOT measure had an acceptable degree of reliability with a reported Cronbach’s alpha of α = 0.79. The advantage of this adapted version is the ease with which data can be gathered as it can be given online and simply requires a multiple-choice response. Direct comparisons to the original paper/pencil PTSOT task (e.g., Hegarty & Waller, 2004; Kozhevnikov & Hegarty, 2001) are difficult to make given the original test required participants draw an arrow from the center of the circle to the target third item in the array. In the original task, the dependent variable was the average absolute deviation between participant’s response and the correct direction to the target. Thus, means reported in the literature for the original PTSOT are a reflection of the participant’s average deviation rather than correct number of items, as was used in the current study. One thing we do see across both the original PTSOT and the adapted PTSOT is individual differences in performance such that there is variability in scores. Future research will need to evaluate whether the adapted PTSOT and the original PTSOT are comparable by using a within-subjects design where participants complete items on both versions and comparisons in performance are made.

The current findings suggest a role for only one affective factor, confidence, in potentially explaining individual differences in two types of spatial ability, mental rotation and perspective-taking/spatial orientation. Contrary to our predictions, no evidence was found in our study to suggest that spatial anxiety, either small or large scale, is related to spatial ability. This lack of relation was unexpected given prior literature showing that individuals who report high spatial anxiety perform worse on mental rotation and navigation tasks (Alvarez-Vargas et al., 2020; Arrighi & Hausmann, 2022; Cooke-Simpson & Voyer, 2007; Desme et al., 2024; Estes & Felker, 2012; He & Hegarty, 2020; Hund & Minarik, 2006; Lawton, 1994; Lyons et al., 2018; Ramirez et al., 2013). Why were we not able to replicate this prior research showing a role for spatial anxiety in explaining individual differences in spatial ability?

One likely explanation for the failure to replicate and extend findings on spatial anxiety and spatial ability could be the measures and/or procedure we used to assess both spatial anxiety and spatial ability. This explanation seems plausible for the lack of relation found between spatial anxiety and perspective-taking since we adapted the perspective-taking measure to be given online and as a multiple-choice assessment. By doing so, we may have changed the nature of this task and how it is solved. By adapting the measures to allow participants to complete them via the computer screen, we may have been inadvertently constraining their position such that they could not employ perspective-taking strategies used in real-life scenarios. Further, we also used a relatively new measure of spatial anxiety that includes not only items about navigation/wayfinding anxiety, but items about mental rotation anxiety. While Alvarez-Vargas and colleagues (2020) found this new measure relates to individual differences in mental rotation, they did not directly examine links to other types of spatial abilities, including those with links to large-scale spatial ability (e.g., perspective-taking/spatial orientation). It is also plausible that the scenarios asked about in the spatial anxiety measure are not directly relevant experiences that lead to the development of strategies to solve the type of perspective-taking task we gave. Perhaps by including spatial anxiety questions that pertain specifically to reorienting in an environment or taking another’s perspective would show relations to perspective-taking ability. Lastly, we may have inadvertently selected a sample of participants who were more homogeneous in their spatial anxiety and/or who were lower in spatial anxiety overall. While we do not have any direct evidence for this claim, it is possible that students completing introductory STEM courses may be less spatially anxious individuals compared to those who choose non-STEM courses (e.g., courses in humanities and liberal arts). More research is needed to determine whether this is the case.

We also think there may be a more mechanistic explanation for our failure to replicate prior links between spatial anxiety and spatial ability. There is some reason to believe that the link between spatial anxiety and spatial ability is moderated by working memory (e.g., Eysenck & Calvo, 1992; Owens et al., 2014; Ramirez et al., 2012), such that the link is only seen in those with better working memory or where there are higher working memory demands (e.g., Ashcraft & Kirk, 2001; Eysenck et al., 2007). Since the need for working memory can vary as task demands increase or decrease, we speculate that our *untimed* spatial measures were less demanding, requiring fewer working memory resources. Thus, it is plausible that our methodological choice to use untimed tasks resulted in fewer working memory resources, which in turn led to a failure to find the link between spatial anxiety and spatial ability. This hypothesis will need further testing in future research where we use timed tasks and also gather data on participant working memory.

The current study is not without its limitations. First, our study was a correlational study. As a result, we cannot say anything about direction of the relations we reported, such that we are unable to say that it is confidence that leads to changes in spatial ability or vice versa. Second, as we mentioned above, our sample of participants included only college students who had completed some introductory STEM college courses. Thus, our findings may not be generalizable to other samples, including college students outside of STEM degree-seeking programs. Further, we only gathered data on 100 participants. Prior work by Hegarty and Waller (2004) and Cooke-Simpson and Voyer (2007) suggested we would be sufficiently powered to detect effects. However, it is possible given some of the uncertainty related to mixed findings in the literature that we were underpowered and unable to detect effects. Third, we only used one measure to assess each construct. This meant should one of our measures have poor reliability or validity that our results may not accurately reflect the true relation between variables. While our psychometric data suggest we had reliable measures and we used existing measures with published reports of high degrees of validity and reliability (e.g., Alvarez-Vargas et al., 2020; Kozhevnikov & Hegarty, 2001; Lawton, 1994; Vandenburg & Kuse), it is still plausible that our null findings were due to measurement issues that could have been illuminated by using more than one measure per construct. Lastly, because we were interested in STEM majors as a broadly defined group, we could not determine whether one major was driving our reported effects, as we had wide variability in the majors that participants had reported and small sample sizes per major.

Future research should continue to explore potential relations between affective factors and individual differences. This future work would benefit from using a larger, less homogenous emerging adult sample that could include both STEM and non-STEM majors. In addition, should future work be interested in examining sex differences in spatial ability and links to affective factors then we advise gathering data on a more balanced sample with equal numbers of males and females and potentially considering gathering information about gender identity. Further investigations are also needed to assess the validity and reliability of the measures used, including the adapted PTSOT and our item-level confidence measure. There is a lot to learn about whether these measures predict a variety of real-life spatial abilities and whether we should be assessing in different ways beyond at the item-level. One should consider alternative approaches to assessing affective factors and spatial ability by including more than one measure per construct and by manipulating the order of assessments or potentially randomizing the order of assessments. Only with these additional data can we be fully confident in the links reported in the literature and in this paper. We also believe that new studies should explore the role of time constraints and gather reaction time data to understand the role of other potential confounding or moderating variables, including stereotype threat and working memory. It may be that other participant characteristic including working memory, stereotype threat, early spatial experiences (e.g., play with spatial toys), and interest in STEM courses, moderate these affective factors and spatial ability relations. Finally, experimental manipulations, as well as longitudinal research, may be better suited to answer lingering questions we have about how individual differences in spatial ability develop over the lifespan and to what degree affective factors cause individual differences in spatial ability.

The current study aimed to explore how to affective factors, confidence and spatial anxiety, relate to variability seen in emerging adults’ spatial ability. Building on prior literature and using established or adapted measures, we found a role for one’s confidence in explaining individual differences in both mental rotation and perspective-taking performance. The results suggest a role for confidence in not just one type of spatial ability but now two types of spatial abilities, both large- and small-scale ability. Future research should explore ways that spatial confidence can be increased and whether confidence as a malleable affective factor can lead to changes in spatial ability. This work could potentially offer a way to improve one’s spatial abilities, an ability that has been tied to STEM achievement (Wai et al., 2009).

## Data Availability

Data and materials are available upon request from the first author.

## Abbreviations

CI: Confidence intervals
FIML: Full information maximum likelihood
MRT: Mental rotation test
M-SAS: Modified spatial anxiety scale
PTSOT: Perspective-taking/spatial orientation task
STAI-T: State-trait anxiety inventory–trait subscale
